# High Frequency Electromagnetic Shielding by Biochar-Based Composites

**DOI:** 10.3390/nano11092383

**Published:** 2021-09-13

**Authors:** Daniele Torsello, Mattia Bartoli, Mauro Giorcelli, Massimo Rovere, Rossella Arrigo, Giulio Malucelli, Alberto Tagliaferro, Gianluca Ghigo

**Affiliations:** 1 Politecnico di Torino, Department of Applied Science and Technology, C.so Duca degli Abruzzi 24, 10129 Turin, Italy; daniele.torsello@polito.it (D.T.); massimo.rovere@polito.it (M.R.); alberto.tagliaferro@polito.it (A.T.); gianluca.ghigo@polito.it (G.G.); 2 Istituto Nazionale di Fisica Nucleare, Sez. Torino, Via P. Giuria 1, 10125 Turin, Italy; 3 Consorzio Interuniversitario Nazionale per la Scienza e Tecnologia dei Materiali (INSTM), Via G. Giusti 9, 50121 Florence, Italy; mauro.giorcelli@polito.it (M.G.); rossella.arrigo@polito.it (R.A.); giulio.malucelli@polito.it (G.M.); 4 Center for Sustainable Future Technologies—CSFT@POLITO, Via Livorno 60, 10144 Torino, Italy; 5 Politecnico di Torino, Department of Applied Science and Technology, Viale Teresa Michel 5, 15121 Alessandria, Italy; 6 Faculty of Science, Ontario Tech University, 2000 Simcoe Street North, Oshawa, ON L1G 0C5, Canada

**Keywords:** biochar, carbon-based composites, electromagnetic shielding, high frequency

## Abstract

We report on the microwave shielding efficiency of non-structural composites, where inclusions of biochar—a cost effective and eco-friendly material—are dispersed in matrices of interest for building construction. We directly measured the complex permittivity of raw materials and composites, in the frequency range 100 MHz–8 GHz. A proper permittivity mixing formula allows obtaining other combinations, to enlarge the case studies. From complex permittivity, finally, we calculated the shielding efficiency, showing that tailoring the content of biochar allows obtaining a desired value of electromagnetic shielding, potentially useful for different applications. This approach represents a quick preliminary evaluation tool to design composites with desired shielding properties starting from physical parameters.

## 1. Introduction

There is an increasing interest in shielding electronic devices and communication instruments against electromagnetic (EM) radiation, in order to improve their efficiency and lifetime. This requirement is sometime extended to entire buildings. In fact, there are particular places that should be well protected against EM interference, e.g., those dedicated to the health sector for applications, such as imaging or tomography, working with microwave- and millimeter-wave devices. In this cases, usually, shielding materials are applied as a coating on wall surfaces. However, the use of shielding elements directly mixed with currently used building materials has been considered only recently, and could represent a sustainable route, especially if the additional materials are eco-friendly and cost-effective [1,2].

The main drawback of EM-shielding materials is related to the drop of mechanical properties due to high filler concentration, as observed by Wen et al. [3]. Accordingly, coating approaches and non structural materials have been used for EM-shielding applications [4]. This opened the way for the production of materials that do not require superior mechanical properties, but only a great shielding efficiency.

The shielding efficiency (SE) of a material depends on its intrinsic properties, i.e., conductivity σ and permittivity ε. Thus, due to high σ, metals are well suited for many EM-shielding applications, but they also have disadvantages, such as heavy weight [5], corrosion propensity [6], mechanical stiffness, and high cost [7]. When metal coatings become unpractical, one can consider conducting composites, showing light weight, flexibility, and low cost [8,9]. Further advantages of this approach include the tunable shielding response, depending on preparation and/or conducting inclusions concentration, and possibly manufacturing of finished products by coating or 3D printing. Among the materials with these properties, promising for EM-shielding applications, carbon-based composites recently emerged as a reliable solution. In particular, an eco-friendly material derived from waste, matching these requirements, is biochar. Biochar is a carbonaceous material produced by thermal treatment of biomass (e.g., from agricultural or food waste), showing good enough properties to be considered as a practical alternative to the use of other more performing carbon-based materials, such as graphene or carbon nanotubes, which are much more expensive and require complex synthesis [10]. SE of carbonaceous-based composite materials reported in literature (a wide selection of which is shown in Table 1) are deeply affected by the intrinsic properties of the fillers, that dominate over matrix properties. High tech, expensive, fillers, such as carbon nanotubes (for which extensive details can be found in Reference [11]) and nanofibers, showed the best results, but high loading levels of biochar are expected to reach comparable performances for a much cheaper price, as necessary in fields where large volumes are required.

A rich and interesting area of research focuses on understanding the origins of EM losses and shielding in different fillers, in order to optimize and engineer them. Magnetic effects are predominant when metallic inclusions are employed [19], and their efficiency can be engineered by changing shape, size, and encapsulation [20]. In other cases, where no metallic particles are added -as in the present case-, the main contribution to shielding comes from the conductivity of the inclusions in the insulating matrix. In this field, carbon materials have a very important role due to the extremely wide tunability of their properties [21], that makes this class of materials pivotal for applications that range from EM shielding to next-generation sensors and devices [22,23]. In the specific case of biochar, several aspects intervene due to its intrinsically disordered nature and to the large range of graphitization degrees that can be obtained depending on its preparation procedure [24]. However, the main contributions to EM shielding in such materials are expected to stem from migration and hopping conductance [25]. As a result of these mechanisms, it turns out that high-carrier-mobility carbon-based fillers are extremely promising and versatile for shielding composites [26]. In addition to the understanding of the mechanisms of EM losses in specific materials, an important aspect is the possibility to quickly estimate the behavior of a composite starting from the physical properties of its matrix and filler. This is a preliminary step needed to focus experimental efforts mainly on the most promising combinations.

In this work, we discuss about the EM-shielding properties of composites made of biochar inclusions dispersed into non-structural materials of interest for building construction. Cement is one of them, since cement-based materials, e.g., paste, mortar, and concrete, are currently widely employed not only for their mechanical properties but also because they are durable and economical, as well as could be easily applied as high filled layer in pre-existent structures. Other materials potentially useful for coatings in buildings are epoxy resins and high-density polyethylene (HDPE). We performed a microwave characterization of biochar composites based on these materials, yielding their complex permittivity. We evaluated the SE of the measured materials and composites, and of other combinations obtained by suitable mixing rules, allowing us to simulate the electromagnetic behavior of composites as a function of biochar loading.

Overall, the promising potential of biochar as low-cost and eco-friendly material to improve shielding to EM interference in buildings is demonstrated, and the methodology proposed in this work is shown to provide a useful preliminary approach to design composites with desired EM-shielding properties.

## 2. Materials and Methods

### 2.1. Biochar, Matrices, and Composites Preparation

The biochar employed in this study (OSR 550: C 68.9 ± 2.3 wt.%, H 1.82 ± 0.22 wt.%, N 1.59 ± 0.22 wt.%, P 0.29 ± 0.08 wt.%, K 2.86 ± 0.26 wt.%, total ash 19.5 ± 0.9 wt.%, metal content in the ppm range) is derived from oil-seed and was purchased from UK Biochar Research Center, where it was produced using a pilot-scale rotary kiln pyrolysis unit [27] with highest treatment temperature 550 °C. We have shown that biochar powders can be thermally treated to tune their conductivity and permeability, and then dispersed in a suitable matrix to obtain convenient mechanical properties [24,28]. For this reason, the biochar was then annealed at 1500 °C by using a vacuum electric furnace (Pro.Ba., Cambiano, Italy) under argon atmosphere (99.99% purity, controlled pressure 550 mbar) with a heating rate of 150 °C/h, a maximum temperature dwell time of 30 min, followed by cooling to room temperature with the same rate used for heating [29]. Biochar-based epoxy composites were prepared according to Bartoli et al. [30], employing a two components Bis phenol A (BPA) diglycidyl resin system purchased from CORES (Cores epoxy resin, LPL). Biochar was mechanically pulverized and subsequently dispersed into the epoxy monomer (biochar loading: 25 wt.%) using a tip ultrasonicator apparatus (Sonics Vibra-cell) for 15 min, employing a pulsed mode with cycles of 20 s (alternated by 10 s pauses) to allow a better heat diffusion and avoid an excessive temperature rise. Further ultrasonication for 2 min was performed after the addition of the curing agent. Then, the mixture was left into the molds for 16 h at room temperature. A final thermal curing was performed using a ventilated oven (I.S.C.O. Srl “The scientific manufacturer”) at 70 °C for 6 h.

Cement (Portland Type 1 52.5 R) was purchased from Italcementi S.p.A., Bergamo, Italy. Samples were prepared by mixing neat cement with water and cured in water for 24 h at 80 °C in 90% humidity atmosphere.

The employed HDPE has the trade name Lupolen 4261AG from Lyondell Basel. Its main characteristics are: melt flow rate (190 °C/21.6 kg) = 6.0 g/10 min, density = 0.945 g/cm${}^{3}$, and melting temperature in the range 120–140 °C. Specimens were obtained by compression molding, using a laboratory press (Collin Teach Line 200T), working at 220 °C, under a pressure of 100 bar for 2 min.

### 2.2. Biochar Characterization

Raman spectra were collected using a Renishaw inVia (H43662 model, Gloucestershire, UK) equipped with a green laser line (514 nm), in the range from 500 cm${}^{-1}$ to 3500 cm${}^{-1}$. X-ray diffraction (XRD) analyses were performed by using a Panalytical X’PERT PRO PW3040/60 diffractometer, with Cu Kα radiation at 40 kV and 40 mA, Panalytical BV, Almelo, The Netherlands. The spectra were obtained from biochar powder in the 2θ range from 15° to 90° with a step size of 0.013°.

### 2.3. Microwave Characterization

The SE of several biochar-based composites has been assessed starting from a microwave characterization in the frequency range 100 MHz–8 GHz of cement, HDPE and epoxy matrices, and biochar-epoxy composites. This frequency range covers the bands from UHF to C, used for communications, including GPS, Wi-Fi, and Bluetooth devices, mobile phones, and radar systems.

In order to achieve a preliminary characterization of a large set of biochar-based composites, we employed a multi-step approach, summarized here and detailed below: (i) we measured the complex dielectric constant of all the matrices, (ii) we determined the dielectric constant of biochar as an inclusion material (which, in principle, might be different from the starting biochar powders) by measuring several concentrations of a specific composite (epoxy + biochar) and employing a proper mixing rule, and (iii) we applied the same rule (inversely) to deduce the effects of biochar addition to the other matrices.

**Figure 1:**
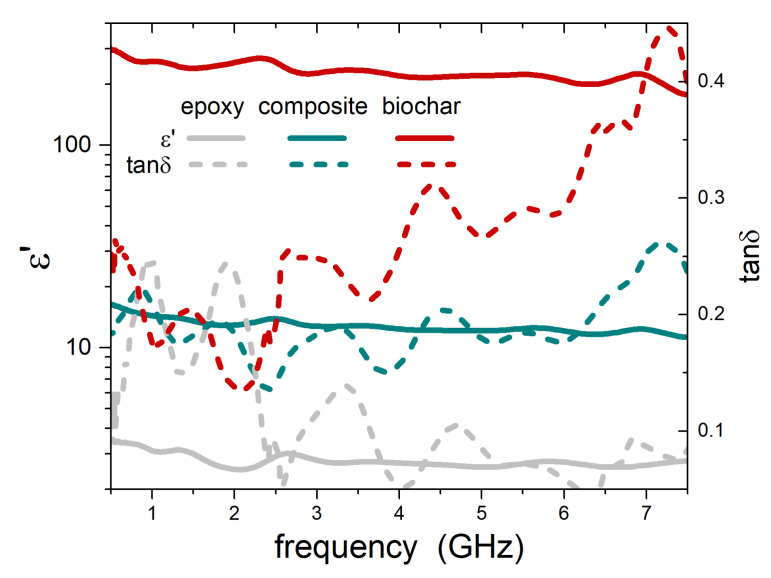
Real part of the permittivity (logarithmic scale, on the left) and loss tangent (linear scale, on the right) for analyzed epoxy and epoxy-25 wt.%-biochar composite (measured), and for biochar inclusions alone (calculated).

The microwave measurements were based on a two-port transmission line technique in a cylindrical coaxial cell (EpsiMu toolkit [31]) connected to a Vector Network Analyzer. The sample was inserted into the cell as a spacer between inner and outer conductors, with diameters of 0.6 and 1.3 cm, respectively. The EM properties of the sample were then extracted by de-embedding and by using a Nicolson-Ross-Weir transmission/reflection algorithm [32,33]. The output of measurements is the complex permittivity of the material, $\varepsilon =\varepsilon^{\prime }-j\varepsilon^{\prime \prime }$, where $j=\sqrt{-1}$. An example is given in Figure 1, where the real part of permittivity $\varepsilon^{\prime }$ and loss tangent $\tan\delta =\varepsilon^{\prime \prime }/\varepsilon^{\prime }$ are shown for epoxy and for an epoxy-25 wt.%-biochar composite. The oscillations in the measured values are due to the geometry of the measurement cell and are common in measurement techniques based on microwave transmission lines. The value of tanδ measured for pure epoxy is in quite good agreement with those commonly reported, especially at higher frequencies where spurious oscillations are smaller. At lower frequencies, the measurement is less precise but still in an acceptable range. Moreover, this uncertainty has a quite small effect on the conclusions drawn in this study, that are based on general trends and not on specific low frequency values.

Once the properties of matrix and composite are obtained, it is also possible to extract the EM properties of the biochar inclusions alone, by using a proper mixing rule, expressing the overall permittivity ε as a function of the complex permittivity of each constituents ($\varepsilon_{m}$ for the matrix and $\varepsilon_{b}$ for biochar inclusions) and volume fraction *f* of the filler [24]. To this aim, several approaches have been reported, depending on the shape of inclusions. We used the Looyenga rule, which does not involve any assumption about the geometry of the inclusion particles [34], and that demonstrated a very good matching with experimental data in similar cases [35]:

(1) $$\varepsilon^{1/3}=\left(1-f\right)\varepsilon_{m}^{1/3}+f\varepsilon_{b}^{1/3}.$$

Figure 1 also reports $\varepsilon^{\prime }$ and tanδ for biochar, resulting from the application of Equation (1). The very high value of $\varepsilon^{\prime }$ for biochar is remarkable (note the log scale) and promising for shielding applications. This result was, in turn, used to calculate, by Equation (1), the complex permittivity of other composites, mixing the measured permittivity of various matrices (cement, HDPE) and the calculated permittivity of biochar. Finally, all these data allowed us to calculate the shielding efficiency, according to the approach described in the following paragraph.

### 2.4. Electromagnetic Shielding Efficiency

**Figure 2:**
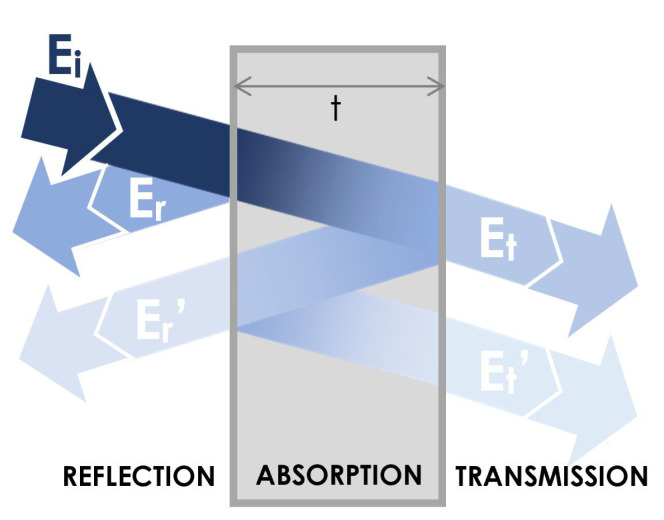
Mechanisms of attenuation of an electromagnetic radiation of energy $E_{i}$, incident on a shielding layer of thickness *t*: reflection, multiple reflections, and absorption. $E_{r}$($E_{t}$) is the reflected (transmitted) energy, and $E_{r}$’($E_{t}$’) is the energy reflected (transmitted) after multiple crossing the layer. Color attenuation represents attenuation of energy.

For an electromagnetic wave propagating into a material with negligible magnetic properties and under the far field conditions (kr≫1, where *k* is the wave number and *r* is the source-detector distance), the overall shielding efficiency SE is expressed, in decibels (dB), as [36,37]:

(2) $$SE=10\;log_{10}\left(\frac{P_{i}}{P_{t}}\right)=SE_{A}+SE_{R}+SE_{MR},$$

where $P_{i}$ and $P_{t}$ are the incident and transmitted power, $SE_{A}$, $SE_{R}$, and $SE_{MR}$ are the absorption, reflection, and multiple-reflection contributions to the shielding efficiency, respectively (see the scheme reported in Figure 2),

(3) $$SE_{A}=20\;\alpha t\;log_{10}e=8.686\;\alpha t\;,$$

(4) $$SE_{R}=20\;log_{10}\frac{{\left|1+n\right|}^{2}}{4|n|}\;,$$

(5) $$SE_{MR}=20\;log_{10}\left|1-exp\left(-2\gamma t\right)\frac{{\left(1-n\right)}^{2}}{{\left(1+n\right)}^{2}}\right|\;,$$

(6) $$\alpha =\frac{2\pi }{\lambda_{0}}\sqrt{\frac{\left|\varepsilon^{\prime }\right|\left(\sqrt{1+\tan^{2}\delta }\mp 1\right)}{2}}\;,$$

(7) $$n=\sqrt{\frac{\left|\varepsilon^{\prime }\right|\left(\sqrt{1+\tan^{2}\delta }\pm 1\right)}{2}}+j\sqrt{\frac{\left|\varepsilon^{\prime }\right|\left(\sqrt{1+\tan^{2}\delta }\mp 1\right)}{2}}\;,$$

*t* is the thickness of the shield, α is the attenuation constant (equal to the inverse of the skin depth), *n* is the complex refractive index, $\gamma =\left(1+j\right)\alpha$ is the propagation constant, $\tan\delta =\frac{\varepsilon^{\prime \prime }}{\varepsilon^{\prime }}$, and upper and lower signs are for positive and negative $\varepsilon^{\prime }$, respectively. It should be noted that all these contributions to the SE are relevant when the thickness of the EM shield is smaller than the skin depth; otherwise, there is no thickness dependence. For the biochar composites investigated in this study, this condition is always satisfied (e.g., for an epoxy-biochar 25 wt.% composite, at 5 GHz, the skin depth is 31 mm [24]). To be complete, it should also be mentioned that SE could be calculated with a similar setup directly from the measurement of the scattering parameters. However, in order to employ the mixing rule and estimate the properties of other composites, we are forced to work with physical parameters of the materials rather than properties of the particular sample/device.

**Figure 3:**
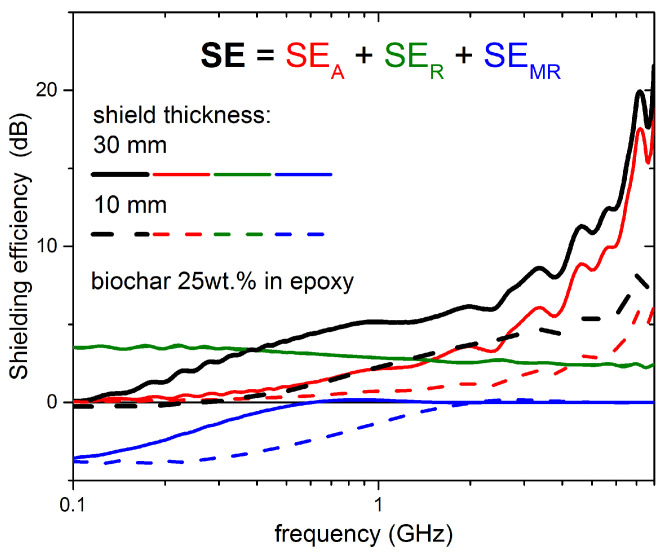
Absorption (A, shown in red), reflection (R, shown in green), and multiple reflections (MR, shown in blue) components of the overall shielding efficiency SE (shown in black), for biochar-epoxy layers of thickness 10 mm and 30 mm. SE was calculated from experimental data shown in Figure 1 (epoxy-25 wt.%-biochar composite, with biochar treated at 1500 °C).

Figure 3 shows the contribution of absorption, reflection, and multiple reflections to the shielding efficiency SE, in the case of biochar-epoxy shielding layers 10 or 30 mm thick, calculated starting from the experimental data shown in Figure 1. It can be noticed that the contribution of the multiple-reflections term can be positive or negative but, generally, negligible when $SE_{A}\geq 10$ dB.

## 3. Results and Discussion

**Figure 4:**
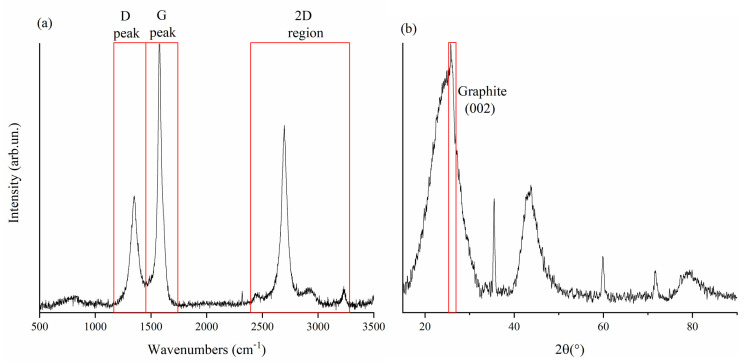
(**a**) Raman and (**b**) XRD spectra of biochar used as filler.

Biochar used as filler for this study was preliminary analyzed by using Raman and XRD spectroscopy, as reported in Figure 4. According to Ferrari et al. [38], the Raman spectra of disordered carbon could be a helpful tool for the evaluation of the disorder of carbonaceous material. In Figure 4, the Raman spectra of biochar used as filler in this work is reported, showing D and G peaks centered at 1347 cm${}^{-1}$ and 1574 cm${}^{-1}$, respectively, and the ratio between their areas (ID/IG) reaches up to 0.78 [29]. Furthermore, the 2D region is very well defined, suggesting the presence of a quite ordered material [39]. XRD spectra (Figure 4b) showed a broad band between 16° to 32° due to the presence of amorphous carbon with a narrow feature at 26° due to the presence of graphite, and another broad signal between 42° and 47° due to the sp${}^{2}$ carbon network. According to previously reported data, biochar used as filler shows graphitic crystallites in-plane size of up to 2.3 nm with an inter-layer spacing of up to 0.344 nm very close to the one of the graphite (0.355 nm) [40]. These analyses clearly show a material not yet fully graphitized but presenting an appreciable amount of graphitic domains. As reported in Reference [41], the carbon structure of biochar treated at 1500 °C was characterized by a high conductivity and could be easily used to improve the conductivity of related composites. Most likely, this increased conductivity (given by a combination of migration and hopping conductance [25] promoted by the ordering of graphitic crystallites), together with interfacial polarization [42], is the main mechanism contributing to EM shielding in biochar composites at these frequencies [43].

**Figure 5:**
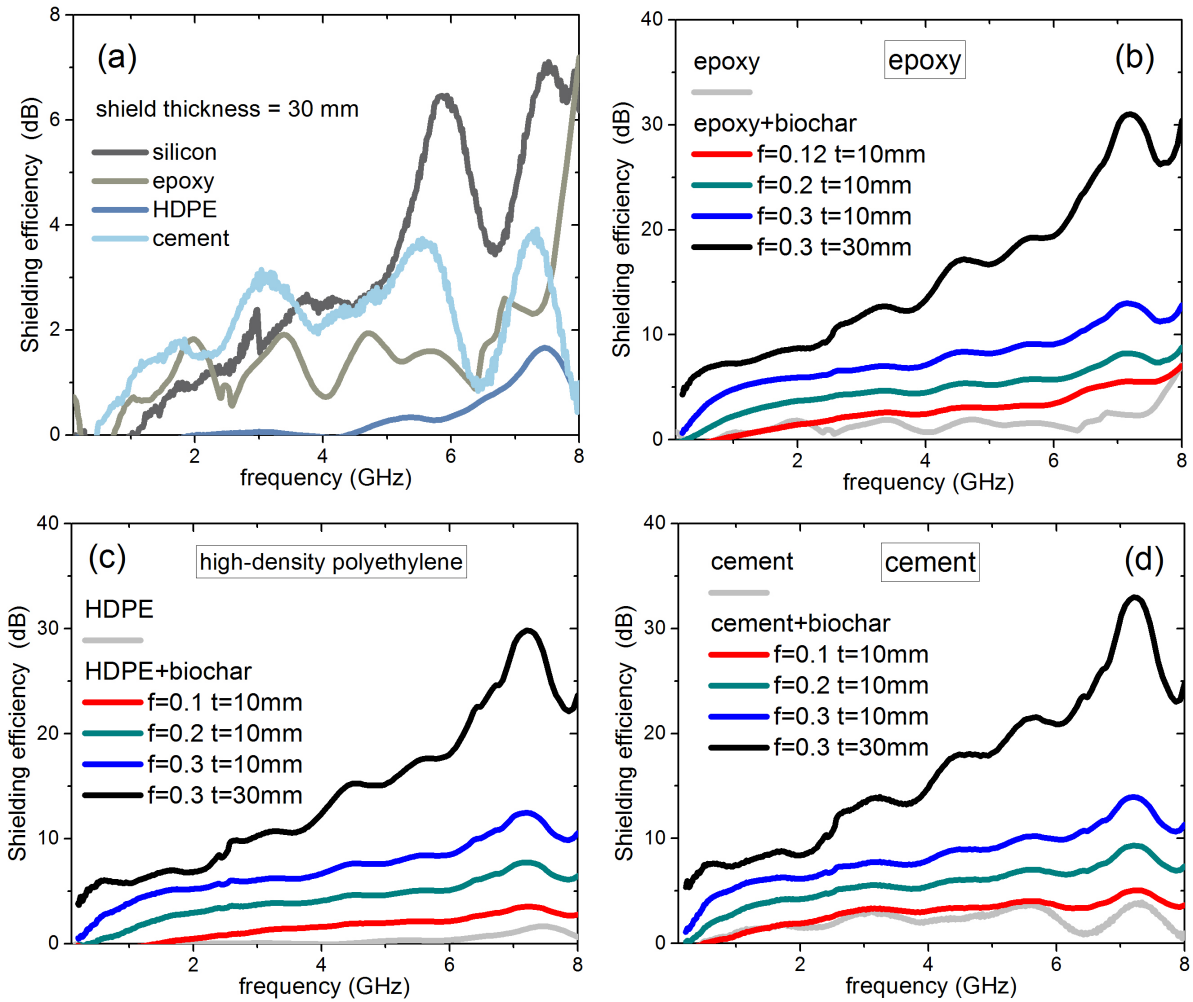
(**a**) Shielding efficiency of a 30-mm-thick uniform layer of different materials: silicon, epoxy, HDPE, and cement. (**b**) Shielding efficiency of a 30-mm-thick uniform layer of epoxy (light gray), of 10-mm-thick epoxy-biochar layers, with different biochar volume fractions (from *f* = 0.12, red, to *f* = 0.3, blue), and of a shield with thickness 30 mm and *f* = 0.3 (black). (**c**,**d**): same as (**b**), but for HDPE and cement.

**Figure 6:**
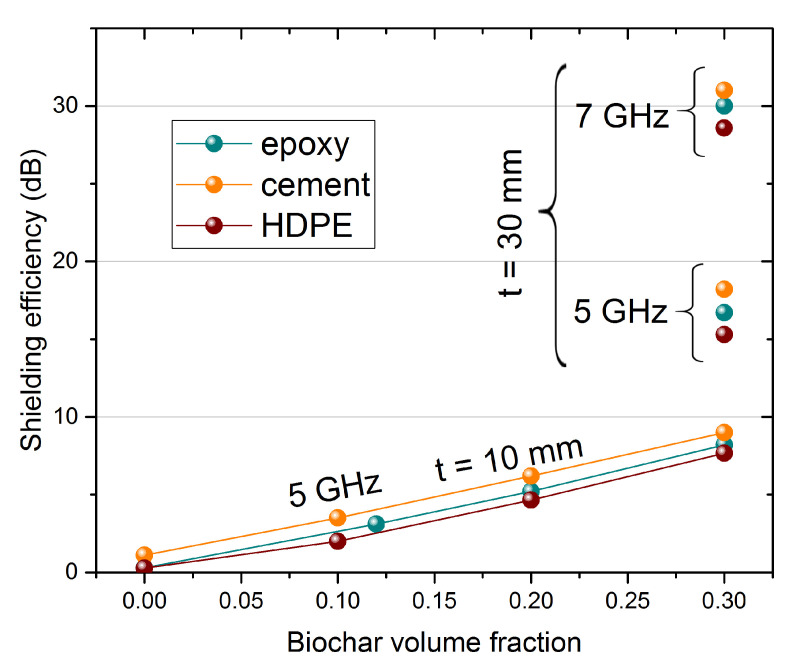
Shielding efficiency for different composites as a function of biochar volume fraction, at the frequencies of 5 and 7 GHz for 10-mm-thick and 30-mm-thick shields. Reference SE values are marked by horizontal gray lines, at 10, 20, and 30 dB, corresponding to 90%, 99%, and 99.9% of shielded EM power, respectively.

In Figure 5a–d, we report calculated SE for various materials and biochar composites, of potential interest as building materials. Figure 5a shows the EM shielding capability of 30-mm-thick layers of silicon, epoxy, HDPE, and cement, obtained starting from direct microwave measurements. Cement shows a larger SE than other matrices, mostly due to a larger real part of the permittivity, with values close to 5 instead of 2–3, whereas the tanδ are comparable for all four materials. It is clear that none of the materials are able to reach, in the selected frequency range, a useful EM shielding level. On the contrary, the addition of biochar inclusions significantly improves the SE performance. Figure 5b shows the case of epoxy: the curves for biochar volume fraction *f* = 0.12, and 0.2 have been calculated directly from experimental data, while the *f* = 0.3 curve has been deduced from data of epoxy and biochar by the mixing rule formula. In Figure 5c,d, similar curves are shown for HDPE and cement, all calculated from data of blank material and biochar-composites by the mixing formula. As expected, the increase of both thickness and biochar content largely enhances the SE of the composites. The most efficient combinations are cement-based, due to the better performance of the matrix itself. However, since the main contribution to SE comes from the inclusions, the spread between different composites decreases with increasing filler content. Finally, in Figure 6, we report a summary of SE of composites at the frequencies of 5 and 7 GHz (representatives of the C band), as a function of biochar volume fraction. SE values of 10, 20, and 30 dB are also reported as horizontal lines, as a reference for application requirements. Clearly, such composites can be considered as a valid route to EM shielding, granting the possibility to achieve a desired SE by choosing the suitable matrix, filler content, and thickness. Particularly promising, from the point of view of decreased environmental impact, is the much smaller thickness and, therefore, overall material required by biochar-filled composites, with respect to non-added matrices, to achieve a certain SE.

## 4. Conclusions

In conclusion, we investigated the microwave shielding efficiency of composites, where biochar inclusions are dispersed in materials of interest for building construction. We directly measured raw materials and some biochar composites, obtaining their complex permittivity in the range from 100 MHz to 8 GHz. Then, we extracted the biochar contribution and calculated the properties of other composite combinations by a suitable permittivity mixing formula. From complex permittivity, finally, we calculated the shielding efficiency, showing that tailoring the content of biochar—a cost-effective and eco-friendly material—and layer thickness allows obtaining a desired value of EM shielding, among those significant for applications. Moreover, annealed biochar properties could be further improved by selecting low inorganic content feedstocks, that would guarantee an improvement of graphitic domains in terms of size and ordering. In addition, the addition of metallic particles could be a viable approach to further increase the SE, in light of the fact that, in the case of biochar, these species could be anchored directly to the filler surface though carbothermal processes. Finally, this mixed experimental and computational approach represents a quick and cost-effective way to explore new solutions for EM shielding and materials optimization in the construction field.

## Figures and Tables

**Table 1:** Representative values of SE of different composites with carbonaceous fillers.

Material	Thickness (mm)	SE (dB)	Frequency (GHz)	Ref.
Flexible graphite flakes	not reported	130	1.0	[12]
15 wt.% of Carbon nanotubes into ABS matrix	1.1	50	8.2	[13]
15 wt.% of carbon nanofibers into ABS matrix	1.1	35	8.2	[13]
15 wt.% of carbon black into ABS matrix	1.1	21	8.2	[13]
18 wt.% of carbon black into EVA matrix	2	18	8.2	[14]
3 layers of epoxy laminated carbon fibers	3	21	1.0	[15]
0.4 wt.% of carbon fibers in cementitious matrix	180	42	1.0	[16]
8 wt.% of commercial biochar in cementitious matrix	180	15	10.0	[2]
20 wt.% of sludge biochar in cementitious matrix	180	10	10.0	[17]
80 wt.% of biochar in UHDPE matrix	140	49	5.0	[18]
